# An Updated Systematic Review on Remdesivir’s Safety and Efficacy in Patients Afflicted With COVID-19

**DOI:** 10.7759/cureus.43060

**Published:** 2023-08-07

**Authors:** Mohamed Yasir, Chetan Reddy Lankala, Pravin Kalyankar, Angela Ishak, Mario Mekhail, Cristina Sestacovschi, Elias Kima

**Affiliations:** 1 Division of Research, California Institute of Behavioral Neurosciences and Psychology, Fairfield, USA; 2 Department of Internal Medicine, Uzhhorod National University, Uzhhorod, UKR; 3 Department of Internal Medicine, Fortis Escorts Hospital, Faridabad, IND; 4 Division of Research and Academic Affairs, Larkin Community Hospital, Miami, USA; 5 Department of Internal Medicine, New York University (NYU) Langone Long Island Community Hospital, New York, USA

**Keywords:** covid-19, antiviral drugs, sars-cov-2 treatment, efficacy, remdesivir

## Abstract

Globally, the coronavirus disease 2019 (COVID-19) had a significant impact on everyone’s lives and put a tremendous strain on healthcare systems. Since the outbreak began, remdesivir has been investigated as a potential treatment for COVID-19 that may be both effective and safe. Remdesivir has had a huge impact on the disease’s progression, complications, and mortality. This review provides an updated assessment of the literature regarding remdesivir’s efficacy and safety for the treatment of patients with COVID-19. The search was performed through PubMed, Web of Science, Cochrane, and Scopus for articles published from 2019 to September 20, 2022. Studies that assessed remdesivir’s efficacy and safety were included in this review, with clinical improvements as the primary outcome measure. Seventeen studies were identified following the implementation of the search strategy. Among them, 11 corroborated remdesivir’s efficacy. Meanwhile, the remaining six studies did not observe a statistically significant difference in clinical improvement. Remdesivir is a potentially safe and effective antiviral that shows clinical improvement especially when used during the early course of the disease. However, current literature still questions its safety in patients who are afflicted with the complications of COVID-19, highlighting the need for studies on a large scale.

## Introduction and background

The severe acute respiratory syndrome coronavirus 2 (SARS-CoV-2) is the cause of coronavirus disease 2019 (COVID-19), which has had a huge impact on the lives of everyone on a global scale and resulted in a drastic burden on healthcare systems worldwide. This pandemic has resulted in the investigation of numerous prospective therapeutics for treating and managing this viral disease. Remdesivir has been highlighted as a potentially effective and safe medicine. As a result, remdesivir was the first and foremost drug to gain approval from the United States Food and Drug Administration (FDA) to treat the severe form of the disease [1]. Animal models of this antiviral demonstrated promising efficacy against both severe acute respiratory syndrome coronavirus 1 (SARS-CoV-1) and the Middle East respiratory syndrome coronavirus (MERS-CoV); hence, the drug was proposed to treat COVID-19 patients amid the ongoing pandemic [2,3]. During initial investigations, remdesivir was noted to be very promising. Furthermore, it was one of the first medications that demonstrated activity against SARS-CoV-2 in vitro, raising expectations for its potential as the desired antiviral therapy [4]. Nevertheless, further elucidation of remdesivir’s pharmacokinetics and pharmacodynamics remains indispensable. For instance, its impact on the disease’s progression, complications, and mortality requires auxiliary investigation. In addition, the drug’s safety and behavior in patients with comorbidities remain controversial [5]. After the approval of the FDA, several reported adverse effects have been seen from using remdesivir in hospitalized patients. In addition, the Solidarity World Health Organization (WHO) trials conducted in 30 countries revealed that remdesivir does not significantly decrease the mortality rate in COVID-19 patients [1]. To address these concerns appropriately, novel, large-scale studies must be conducted, and relevant data collection and analysis must be performed. Given this pandemic’s extensive impact on physical, mental, and social well-being, various studies were conducted simultaneously, providing substantial information in a relatively short period of time. This systematic review will examine current literature to provide detailed information on the safety profile of remdesivir as well as its efficacy and address any concerns raised in managing moderate to severe forms of the illness.

## Review

Methods

Search Strategy and Data Extraction

The Preferred Reporting Items for Systematic Reviews and Meta-Analyses (PRISMA) 2020 guidelines were followed for conducting this systematic review [6]. PubMed, Web of Science, Cochrane, and Scopus were screened for full-text journal articles published from 2019 to September 20, 2022. We used the Population, Intervention, Control, and Outcome (PICO) model to formulate a research question with Medical Subject Headings (MeSH) terms and keywords (Table 1). Data extraction was conducted on a Microsoft Excel sheet (Microsoft Corp., Redmond, WA, USA), including authors’ names, study designs, countries where studies were conducted, duration of each study, sample sizes, interventions, controls, and findings of each study.

**Table 1:** PICO framework COVID-19: coronavirus disease 2019, PICO: Population, Intervention, Control, and Outcome, SARS-CoV-2: severe acute respiratory syndrome coronavirus 2

Population	“COVID-19” OR “coronavirus” OR “SARS-CoV-2”
Intervention	“Remdesivir”
Comparison	Any other method of approved treatment for COVID-19
Outcome	“Therapeutic Potential” OR “Patient Outcomes” AND “Clinical Improvement”

Eligibility Criteria

We employed the Endnote X9 software (Clarivate Analytics, Philadelphia, PA, USA) to track citations, identify duplicates, and eliminate references during the initial search strategy. The three inclusion criteria favored studies that involved (i) patients with confirmed COVID-19 by reverse transcription polymerase chain reaction (RT-PCR) testing, (ii) patients with confirmed pneumonia and moderate COVID-19 with comorbidities, and (iii) remdesivir and its effects on patients’ clinical outcomes, irrespective of the setting and the patient’s ethnicity, gender, and age. Exclusion criteria included animal studies, narratives or previous literature reviews, non-journal articles, and journal articles originally published in a language other than English.

Assessment of Risk of Bias

Two tools were employed to determine the risk of bias in our review. First, the Revised Cochrane Collaboration tool, Risk of Bias 2 (RoB 2), was used in assessing randomized trials [7]. In contrast, non-comparative and non-randomized comparative studies were assessed using the Methodological Index for Non-Randomized Studies (MINORS) tool [8].

Results

Literature Search

**Figure 1:**
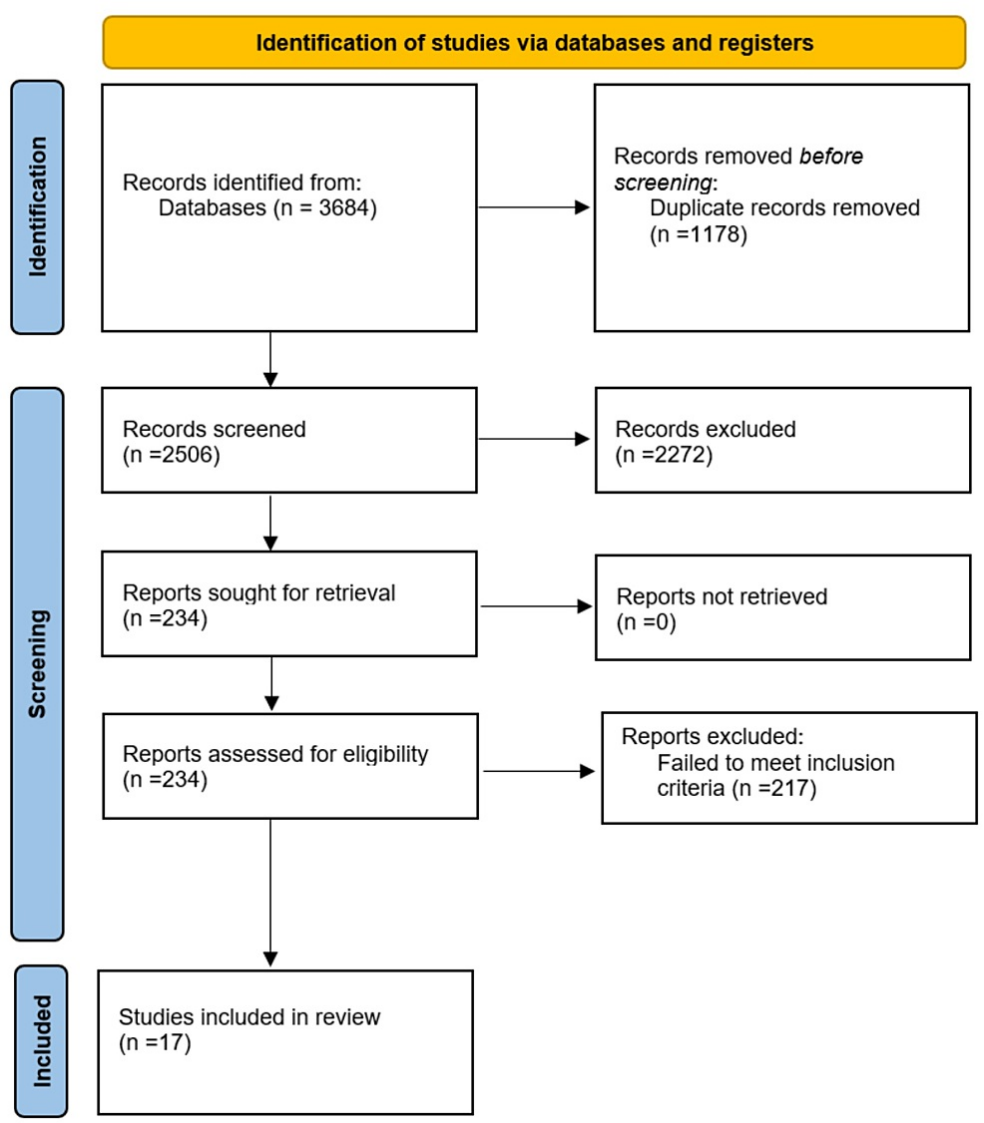
PRISMA flow diagram for screening and selection of studies n: number, PRISMA: Preferred Reporting Items for Systematic Reviews and Meta-Analyses

Our initial search of the databases identified 3,684 individual citations. The elimination of duplicate titles and assessment of preselected articles against the eligibility criteria yielded 17 full-text journal articles, which were included in our final qualitative analysis (Figure 1) [9].

Risk of Bias

**Figure 2:**
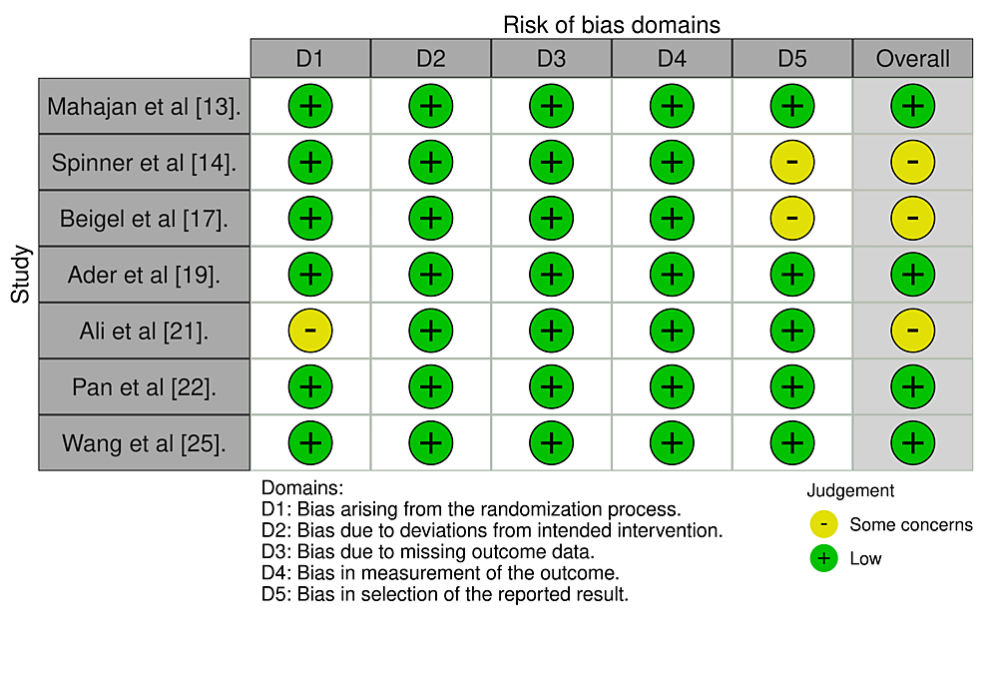
Risk of bias assessment using the Revised Cochrane Collaboration’s RoB 2 tool RoB 2: Risk of Bias 2

**Figure 3:**
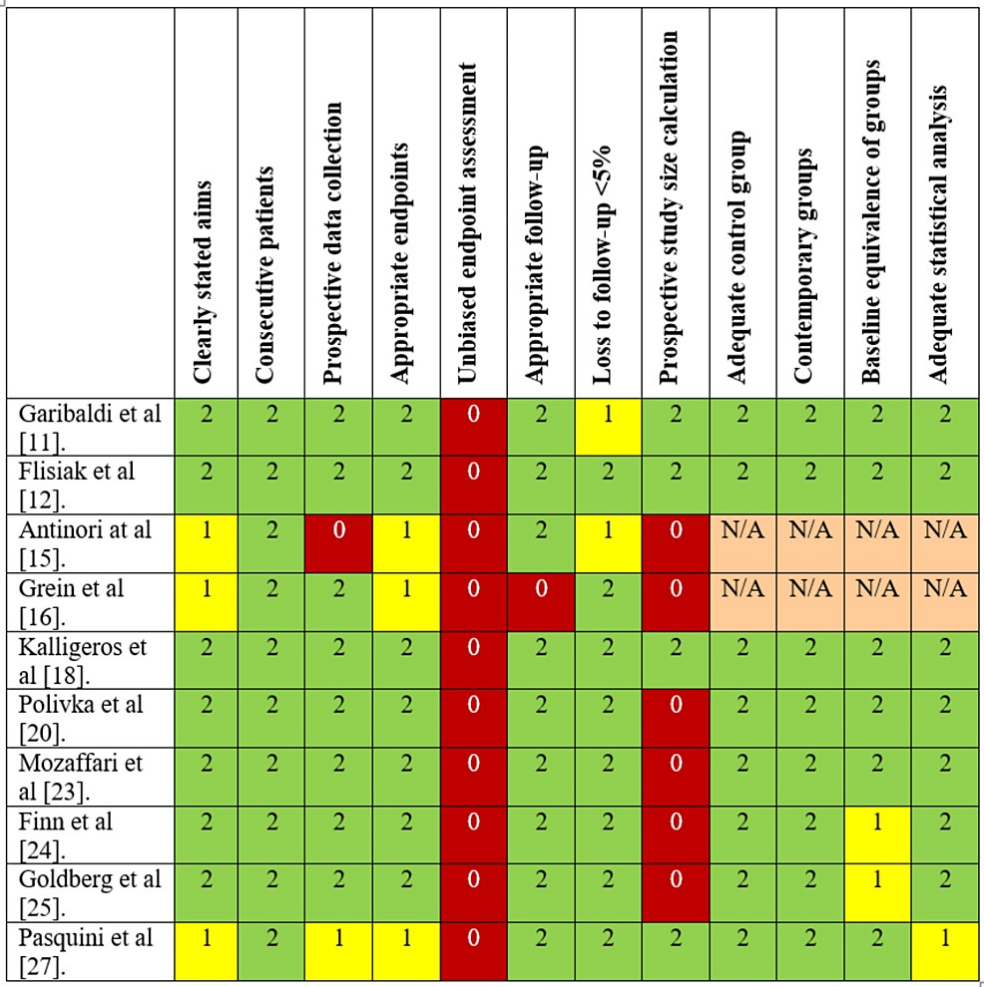
Risk of bias assessment using the MINORS tool MINORS: Methodological Index for Non-Randomized Studies, N/A: not applicable

Out of the seven randomized controlled trials (RCTs), four were considered to have a low risk of bias according to the RoB 2 tool. In contrast, the risk of bias in the other three was considered to have some concerns as there was a deviation from the intended intervention or bias in selecting the reported results (Figure 2) [10]. The overall average MINORS score was 20.1 (median: 20, range: 18-22) for comparative studies and 9.6 (median: 8, range: 7-14) for non-comparative studies. Approximately 25% of the non-randomized studies had a high risk of bias (Figure 3).

Characteristics of the Included Literature

The literature included in this systematic review consisted of both observational and experimental studies (Table 2). While the 11 observational studies included four prospective (one of them randomized) and seven retrospective cohort studies, the six clinical trials were made up of three randomized controlled trials, among which three had placebo controls and three had standard care as their control. The studies were from three geographical regions, mainly the United States of America (USA), in addition to Europe and Asia.

**Table 2:** Characteristics of the included studies USA: United States of America, UK: United Kingdom, N/A: not applicable, COPD: chronic obstructive pulmonary disease

Author (year)	Study design	Country of origin	Duration (months)	Number of patients	Intervention	Control	Findings
Garibaldi et al. (2021) [11]	Retrospective cohort study	USA	6	2,483	Remdesivir (n=158), remdesivir plus corticosteroids (n=184)	Other treatment (n=2141)	Patients receiving remdesivir reached clinical improvement in a short time and had lower 28-day mortality. Corticosteroids with remdesivir did not affect the clinical outcome.
Flisiak et al. (2021) [12]	Retrospective cohort study	Poland	6	333	Remdesivir	Lopinavir/ritonavir	Patients receiving remdesivir had more remarkable clinical improvement and lesser adverse effects, especially in those with oxygen saturation of <95%.
Mahajan et al. (2021) [13]	Prospective randomized cohort study	India	7	82	Remdesivir (n=34)	Standard care (n=36)	There were no statistically significant differences in clinical outcomes.
Spinner et al. (2020) [14]	Randomized clinical trial	USA, Europe, Asia	1	584	Remdesivir 10-day course (n=197), five-day course (n=199)	Standard care (n=200)	Patients who received a five-day course of remdesivir experienced a better clinical outcome than those receiving standard care. There was no statistically significant difference in clinical outcome in patients on a 10-day course of remdesivir. Patients on remdesivir experienced more adverse effects than those on standard care.
Antinori et al. (2020) [15]	Prospective cohort study	Italy	1	35	Remdesivir (n=24)	N/A	Remdesivir improved clinical outcomes in patients admitted to general wards and not in Intensive Care Unit patients.
Grein et al. (2020) [16]	Prospective cohort study	USA, Europe, Canada, Japan	N/A	61	Remdesivir (n=53)	N/A	Patients receiving remdesivir had a decrease in supplemental oxygen requirements.
Beigel et al. (2020) [17]	Randomized, double-blind, placebo-controlled trial	USA, Denmark, UK, Greece, Germany, Korea, Mexico, Spain, Japan, Singapore	3	1,062	Remdesivir (n=541)	Placebo (n=521)	Patients who received remdesivir had a shorter recovery time, a lower mortality rate at days 15 and 28, and lesser adverse effects.
Kalligeros et al. (2020) [18]	Prospective cohort study	USA	4	224	Remdesivir (n=99)	Supportive care (n=125)	There was no statistically significant difference in clinical outcome. Remdesivir had no effect on the development of acute kidney injury.
Ader et al. (2022) [19]	Randomized controlled trial	Europe (France, Belgium, Austria, Portugal, Luxembourg)	10	857	Remdesivir plus standard care	Standard care	No clinical benefit was observed from the use of remdesivir in patients who were admitted to the hospital for COVID-19, were symptomatic for more than seven days, and required oxygen support.
Polivka et al. (2022) [20]	Retrospective cohort study	Hungary	7	947	Remdesivir and supplemental oxygen	Standard care	Hospitalized COVID-19 patients with five-day remdesivir treatment had significantly lower 30- and 60-day all-cause mortality, despite their more severe clinical condition. Men and patients with multiple comorbidities, including COPD, profited the most from remdesivir treatment in the long term.
Ali et al. (2022) [21]	Randomized controlled trial	Canada	8	1,282	Remdesivir (n=634)	Standard care (n=648)	Remdesivir, when compared with standard care, has a modest but significant effect on outcomes important to patients and health systems, such as the need for mechanical ventilation.
Pan et al. (2021) [22]	Randomized controlled trial	30 countries	N/A	11,330	Remdesivir (n=2,750), hydroxychloroquine (n=954), lopinavir (n=1,411), interferon (n=1,412), interferon plus lopinavir (n=651)	Placebo (n=4,088)	There were no statistically significant differences in clinical outcomes.
Mozaffari et al. (2022) [23]	Retrospective cohort study	USA	4	76,046	Remdesivir (n=34,230)	N/A	Remdesivir was associated with a reduction in mortality at 14 and 28 days. This mortality benefit was also seen for no supplemental oxygen, low flow oxygen, and invasive mechanical ventilation/extracorporeal membrane oxygenation at 14 and 28 days.
Finn et al. (2022) [24]	Retrospective cohort study	USA	9	2,062	Remdesivir (n=752)	N/A	Patients were less likely to be readmitted within 30 days if they received remdesivir; associations were strongest for those with mild disease. Remdesivir treatment was associated with a reduction in all-cause mortality and an increase in length of stay.
Wang et al. (2020) [25]	Randomized, double-blind, placebo-controlled trial	China	1	237	Remdesivir (n=158)	Placebo (n=79)	There was no statistically significant difference in clinical outcomes; patients with symptom duration of less than or equal to 10 days on remdesivir had a faster time to clinical improvement compared to those receiving placebo.
Goldberg et al. (2021) [26]	Retrospective cohort study	Israel	9	142	Remdesivir (n=29)	Other treatment (n=113)	Remdesivir treatment shortened the hospital stay by 3.1 days.
Pasquini et al. (2020) [27]	Retrospective cohort study	Italy	1	51	Remdesivir (n=25)	Standard care (n=26)	Patients receiving remdesivir had lower mortality and higher survival rate than those on standard care.

Although the eligibility criteria did not stipulate any geographical restrictions, data extraction focused on studies conducted on the three continents extensively afflicted by the pandemic, including the USA, Europe, and Asia. There was remarkable variation in the sample sizes, ranging from 35 participants to 11,330. In addition, patients with different comorbid conditions, concomitant medications, and disease severity were included in our review to determine the clinical efficacy of remdesivir. Finally, all the studies assessed clinical improvement by measuring various parameters, including the WHO severity score, the Charlson Comorbidity Index score, time to recovery, decrease in mortality, and presence of adverse effects.

Adverse Effects

Nausea, vomiting, and elevated transaminase levels were the most common side effects reported in patients who were treated with remdesivir [11-16]. Moreover, four studies revealed that patients who were treated with the same drug experienced acute kidney injury (AKI) [11,15-17].

The study by Antinori et al. was conducted among 35 patients, of whom 18 were mechanically ventilated patients in the intensive care unit (ICU) with an oxygen saturation of less than 94% on room air and 17 were admitted to the infectious disease ward. Of the patients, 63% completed the scheduled 10-day remdesivir treatment course. Nine patients from the ICU and four from the infectious disease unit discontinued the treatment after five doses due to toxicity (eight patients), death (four patients), and early discharge (one patient) [15].

Additionally, two studies noted elevated serum creatinine levels in patients taking remdesivir [13,17], mostly in patients aged 70 or older with comorbid conditions. The prevalence of hypokalemia among patients who received remdesivir was 6%, compared to 2% in those who received standard care [14].

However, one study found no statistically significant difference between patients getting supportive treatment and those receiving remdesivir in the development of AKI [18], and one study found no statistically significant difference in the occurrence of adverse effects between the groups [13]. Of note, none of the 17 selected studies reported cardiovascular side effects.

In the DisCoVeRy trial, the investigators attributed three deaths to hepatorenal syndrome, bacterial infection, and acute respiratory distress syndrome in the remdesivir group [19].

The study by Antinori et al. has also shown positive treatment outcomes over 10 days. Six patients were discharged with no oxygen supplementation, and two were hospitalized but did not require hospitalization. The study saw an overall improvement of 88.2% in the ward patients during the 28th day of follow-up, with 14 patients discharged. Patients with a comorbid condition such as hypertension were at an increased risk of developing an AKI. Out of 18 patients in the ICU, eight (22.8%) developed AKI, and seven patients were seen with increased bilirubin levels [15].

Another study by Beigel et al., comprising 53 patients, has shown positive clinical outcomes in managing COVID-19 infection. Out of 53 patients receiving the dose of remdesivir, 40 received the dose for the 10-day course, 10 received remdesivir for a period of five to nine days, and three received the dose for less than five days of treatment. At baseline, 34 patients were on invasive ventilation, and four were on extracorporeal membrane oxygenation (ECMO). Out of 53 patients, 36 demonstrated improvement in their oxygen support after 18 days of follow-up following the first dosage of remdesivir, while eight patients deteriorated even with oxygen support. The notable aspect of the study was that 17 of the 30 patients who were mechanically ventilated (invasive) were extubated, and three patients did not need any more ECMO [17].

In the study by Polivka et al., there was a lower 30- and 60-day all-cause death in hospitalized patients who were treated with remdesivir for five days. Men with WHO ordinal scale 4, a seven or higher Charlson Comorbidity Index (having greater than or equal to seven comorbidities out of 17), those with chronic obstructive pulmonary disease (COPD), and patients without heart failure, anemia, diabetes mellitus, dyslipidemia, coronary artery disease, or bronchial asthma significantly benefited from using remdesivir [20,28,29].

Among 1,282 patients, the need for new mechanical ventilation was 8% in the remdesivir group, while it was twice as high in those who received standard therapy in the study by Ali et al. These patients were not mechanically ventilated at baseline. At day 28, the mean oxygen-free days in those receiving remdesivir were 15.9±10.5 and 14.2±11.3 in those receiving standard of care, while the mean ventilator-free days were 21.4±11.3 in the former and 19.5±12.3 in the latter [21].

In the study by Mozaffari et al., there was a decrease in 14- and 28-day mortality in the remdesivir group, and it was most apparent among patients receiving no ECMO, invasive mechanical ventilation, low-flow oxygen, or supplemental oxygen. A decreased risk of mortality at 14 days was also observed in the remdesivir group patients who received noninvasive ventilation or high-flow oxygen [23].

In another study by Finn et al., remdesivir therapy correlated with a 19% decrease in the likelihood of getting readmitted within 30 days after receiving the drug and a 35% decrease in all-cause mortality the following discharge. Of note, two out of every three patients with mild disease in the remdesivir group were less likely to get readmitted within a month [24].

Discussion

An analog of the naturally occurring adenosine triphosphate (ATP), remdesivir is a prodrug of adenosine nucleotide that is metabolized to remdesivir triphosphate (RDV-TP) intracellularly. This nucleotide metabolite has the potential to inhibit the ribonucleic acid (RNA)-dependent RNA polymerase of SARS-CoV-2. Remdesivir has demonstrated the potential to inhibit RNA polymerases of viruses and has a vast spectrum of activity against several viruses [18]. However, despite FDA approval of this medication for the treatment of COVID-19 in children as well as adults, its use has been controversial. The WHO updated its guidelines in late 2020 advising against the administration of remdesivir in COVID-19 hospitalized patients, irrespective of the severity of the disease [30].

Remdesivir prevents viral DNA replication, lowering the viral load. Current literature suggests that viral load testing is an appropriate method of assessing remdesivir’s effects on SARS-CoV-2 [31]. However, 10 studies included in this systematic review failed to utilize this assessment method. The two studies that performed viral load testing among participants of various groups noted no statistically significant difference in viral loads [25,26].

Regarding the primary outcome measured in this systematic review, multiple studies reported clinical improvement in patients receiving remdesivir. One study noted significant clinical improvement in mechanically ventilated patients [27], while another showed an improvement in 14- and 28-day mortality in this population group [23]. However, five others did not document findings of any clinically significant improvement between patients receiving remdesivir and those in the control groups [11,16,25,27,30]. Thus, the effectiveness of remdesivir remains questionable.

It has been demonstrated that a five-day course of remdesivir may be more beneficial than a 10-day course, although the latter carries a 36% higher risk of an adverse drug reaction. According to the research by Beigel et al., patients who received remdesivir for 10 days required less mechanical ventilation and oxygen supplementation [17]. A reduction in the need for mechanically ventilating the patients was also evident in the research by Ali et al. [21].

The non-randomized studies of the intervention have shown that the 28-day risk of death was reduced by up to 44% in the group treated with remdesivir in comparison with the non-remdesivir group, which potentially reflects its clinical efficacy [32].

The safety profile of remdesivir, like the skepticism surrounding its effectiveness in treating severe COVID-19 infection, warrants further investigation. Many studies showed that remdesivir was relatively safe [12,13,17,18], but two studies reported the contrary [16,33]. Some studies in our review reported adverse effects associated with remdesivir, which seemed to occur mostly in critically ill patients with comorbid conditions.

The Infectious Diseases Society of America (IDSA) revised their recommendations for patients (hospitalized and ambulatory) with mild to moderate COVID-19 and a high risk of progression to severe disease, recommending initiating remdesivir within seven days of onset of symptoms rather than no remdesivir [34].

Remdesivir was the first medication to be approved by the FDA for the treatment of COVID-19 in young children, especially those who were more than 28 days old and weighing more than three kilograms, who were hospitalized or not, and who had a high risk of developing severe COVID-19 [33]. This approval is supported by the CARAVAN study, an ongoing interventional clinical trial [35].

Limitations

This systematic review acknowledges some limitations. First, several potentially relevant clinical trials were excluded from this review, as their final results were still pending when our initial search of articles was conducted. Moreover, the lack of uniform methodology and follow-up between the studies was a setback to the comparability of our findings, as it did not allow for quantitative analysis.

## Conclusions

Remdesivir, an antiviral medication, may benefit patients who are infected with mild or moderate forms of COVID-19 infection. Several research findings have suggested the safety and efficacy of remdesivir for a five- to 10-day course with mild adverse reactions. However, larger RCTs are still required to assess its long-term effects.
